# Consumption Patterns of Milk and 100% Juice in Relation to Diet Quality and Body Weight Among United States Children: Analyses of NHANES 2011-16 Data

**DOI:** 10.3389/fnut.2019.00117

**Published:** 2019-08-08

**Authors:** Matthieu Maillot, Florent Vieux, Colin D. Rehm, Chelsea M. Rose, Adam Drewnowski

**Affiliations:** ^1^MS-Nutrition, Faculté de Médecine la Timone, Laboratoire C2VN, Marseille, France; ^2^Albert Einstein College of Medicine, Montefiore Medical Center, New York, NY, United States; ^3^Center for Public Health Nutrition, University of Washington, Seattle, WA, United States

**Keywords:** milk, 100% juice, SSB, children, diet quality

## Abstract

**Background:** The American Academy of Pediatrics (AAP) has recommended placing limits on the consumption of milk and 100% juice by children.

**Methods:** Consumption data for 9,069 children aged 2–19 years came from three cycles of the nationally representative National Health and Nutrition Examination Survey (NHANES 2011-2016). Beverages were classified into 100% juices, milk (whole, reduced fat, and skim), caloric sugar sweetened beverages (SSB), low calorie beverages (LCB), and drinking water. The Healthy Eating Index 2015 and Nutrient Rich Food Index NRF9.3 were two measures of diet quality. Analyses examined consumption patterns for milk and 100% juice in relation to diet quality, AAP recommendations, and BMI z-scores across time and for different age groups.

**Results:** Intakes of milk and 100% juice declined sharply with age, whereas SSB and water increased. Top quartiles of HEI 2015 and NRF9.3 diet quality scores were associated with higher intakes of water, milk, and 100% juice and with lower intakes of SSB. Lower-income groups drank less skim milk and water and more whole milk and SSB. Only 30% of the children consumed any 100% juice. There was no association between the consumption of milk or 100% juice and BMI z-scores for any age group.

**Conclusions:** Top quartiles of diet quality were associated with more milk, 100% juice, and water, and less SSB. Higher quality diets were associated with lower compliance with the AAP 100% juice recommendations. Compliance with the AAP 100% juice recommendations was not associated with lower body weights. Attempts to limit the consumption of milk and 100% juice by children might have the unintended consequence of increasing consumption of SSB and may have limited value for obesity prevention.

## Introduction

The role of milk and 100% fruit juice in the US children's diets continues to be a topic of debate (1–6). To prevent excess weight gain, the American Academy of Pediatrics (AAP) has recommended that children switch to low-fat or non-fat milk after the age of 2 years (7). The AAP has also set limits on the consumption of 100% fruit juices. The suggested amounts for 100% fruit juice were 4–6 oz./d for children aged 4–6 years and up to 8 oz./d for children aged 7–18 years (8). That would limit 100% fruit juice to only one of the recommended 2 to 2 ½ cups of fruit servings per day. For the remainder of the paper 100% juice will be referred to simply as “juice.”

Past studies have shown that beverage drinking patterns built around milk and juice were more nutrient-rich than beverage patterns built around sugar-sweetened beverages (SSB) (4). However, only a small minority of children drank mostly milk, juice, or both. The consumption of milk and juice dropped sharply with age, to be replaced by SSB. Efforts are under way to replace SSBs along with juices and milk in the children's diets, with non-caloric and non-nutritive plain drinking water.

Replacing one food or one beverage with another can be problematic when consumption patterns fall along a socioeconomic gradient (9). In past studies, consumption patterns for juice, milk, and even tap water showed very different links to socioeconomic status (SES) (10). For example, higher-SES groups consumed more whole fruit, whereas lower-SES groups drank more fruit juice. Economic issues may have been an underlying factor. A modeling study showed that while eating whole fruit cost more, a combination of whole fruit and juice effectively remedied inadequate fruit consumption, without increasing diet cost (11).

However, social gradients in other beverage choices are not always related to cost. Past analyses of NHANES data showed that higher SES groups drank more low-fat milk, whereas lower SES groups drank more whole milk. Higher SES groups drank more diet soda, whereas lower SES groups drank more regular soda. Higher SES groups also drank more plain water from the tap (12–14).

Obesity prevalence in childhood and in adult life also follows a socio-economic gradient (15). We may therefore expect SES-driven consumption of whole fruit, skim milk, and water to be associated with lower obesity rates. Indeed, reducing added sugars by replacing SSB with plain drinking water is a recognized strategy for obesity prevention (16). However, past studies linking beverage consumption to body weight may not have fully accounted for the potential (and mostly unobserved) SES confounds. Whereas, obesity has been viewed as the direct consequence of eating a specific food or beverage, it can also be viewed as the outcome of eating patterns associated with lower education and lower incomes (17).

This study examined consumption patterns for milk, fruit juice, and water in a representative sample of toddlers, children, and adolescents in the US. The primary goal was to examine the relation between beverage consumption patterns and diet quality measures. A secondary goal was to examine any links between the consumption of milk and juice and body weight by age, within the confines of a cross-sectional study design.

## Materials and Methods

Dietary intakes data for 9,069 children aged 2–19 years came from the first day of the National Health and Nutrition Examination Survey (2011-2016 NHANES). The NHANES 24-h recall uses a multi-pass method, where respondents reported the types and amounts of all food and beverages consumed in the preceding 24-h, from midnight to midnight. The multi-pass method is conducted by a trained interview using a computerized interface. The recall first identifies a quick list of foods and beverages consumed. The time and occasion for each food item is also obtained. A detailed cycle is then conducted that records the amounts consumed, followed by a final probe for any frequently forgotten foods (beverages, condiments). Day 1 interviews were conducted by trained dietary interviewers in a mobile examination center. For children of 4-5 years, the dietary recall was completed entirely by a proxy respondent (i.e., parent or guardian with knowledge of child's diet). Children aged 4–11 years were the primary respondents, but a proxy respondent was present and able to assist. Children aged 12–19 years were the primary source of dietary recall information, but could be assisted by an adult who had knowledge of their diet.

### Participant Characteristics

NHANES participants were stratified by gender and age. The sample was stratified by age into toddlers (2–4 years); young children (5–8 years); older children (9–13 years); and adolescents (14–19 years). These age groups generally correspond to the age groups used by the IOM (18). The cut-points for the family income-to-poverty ratio were: <1.3, 1.3–1.849, 1.85–2.99, and ≥3.0. Information from the demographic NHANES questionnaires was used to stratify the sample by race/ethnicity, defined as Non-Hispanic White; Non-Hispanic Black, Mexican American, Other Hispanic, Non-Hispanic Asian, and other/mixed race (19). The ethics board review for the NHANES data collection is documented by the National Center for Health Statistics online (20). Analyses of publicly available federal NHANES data are exempt from approvals by Institutional Review Boards.

### Beverage Consumption Patterns

Beverages were classified into 6 categories as follows: (1) fruit juices (citrus juices, apple juice, and non-citrus juices) and vegetable juices. (2) Milk and milk beverages, separated into whole, reduced fat, and skim. (3) Other caloric sugar sweetened beverages or SSB (>50 kcal/240g). (4) Other non-caloric and low-calorie beverages or LCB (<50 kcal/240g). (5) Drinking water, separated into tap and bottled. (6) Baby formula. The 100% fruit juice blends (e.g., apple-cranberry) were included in the juice category but sweetened fruit-based drinks with added sugars were placed among SSBs. All milk analyses were stratified into 3-levels: whole, reduced, and low-fat/skim. Baby formula was included, even though its consumption was low. Very few toddlers> 2 years old consumed breast milk.

### Measures of Diet Quality

Energy and nutrient intakes for NHANES participants were calculated using the Food and Nutrient Database for Dietary Studies 2011-2014, customized with the addition of vitamin D and added sugars. This information was supplemented with data from the Food Patterns Equivalents Database (FPED) from the United States Department of Agriculture (USDA) (21).

The HEI-2015 is the latest iteration of the USDA diet quality measurement tool, specifically designed to monitor compliance with the 2015 Dietary Guidelines for Americans (22). The HEI-2015 is a 100-point scale where the adequacy components are fruits (10 points), vegetables (10), grains (10), dairy (10), protein foods (10), and fats (10). The HEI 2015 adequacy component is based around food groups to encourage, with some food categories called out by name (i.e., total vegetables, dark-green and orange vegetables, total fruit, whole fruit, whole grains, total protein foods, protein from seafood and plant sources, the ratio of polyunsaturated and monounsaturated fatty acids to saturated fatty acids, and total dairy). The moderation component listed foods and nutrients to limit that included refined grains, sodium added sugars and saturated fats. Former versions listed SoFAAs, a composite of solid fat, alcohol, and added sugars that served as a summary measure of empty calories.

The Nutrient Rich Foods (NRF) index served as the second measure of dietary nutrient density (23–25). The NRF9.3d variant is an energy-adjusted diet quality score that is based on 9 nutrients to encourage and 3 nutrients to limit. Reference daily values (DVs) were based on the US Food and Drug Administration (FDA) and other standards (26). The qualifying nutrients to encourage and standard reference amounts were as follows: protein (50 g), fiber (28 g), vitamin A (900 RAE), vitamin C (90 mg), vitamin D (20 mcg), calcium (1300 mg), iron (18 mg), potassium (4,700 mg) and magnesium (420 mg). The 3 disqualifying nutrients and maximum recommended values (MRVs) were: added sugar (50 g), saturated fat (20 g) and sodium (2,300 mg). The NRF was calculated as follows.

$$\begin{array}{l} \text{NRF}\;9.3\text{d}=\left(\text{NR}-\text{LIM}\right)\;\times 100 \end{array}$$

with

$$\begin{array}{l} NR=\overset{9}{\underset{i=1}{\sum }}\frac{Intake_{i}/Energy\times 2000}{DV_{i}} \end{array}$$

and

$$\begin{array}{l} LIM=\overset{3}{\underset{i=1}{\sum }}\frac{Intake_{i}/Energy\times 2000}{MRV_{i}}-1 \end{array}$$

where intake_i_ is the daily intake of each nutrient i, and DVi is the reference daily value for that nutrient. In NR calculation, each daily nutrient intake i was adjusted for 2000 kcal and expressed in percentage of DV. Following past protocol, percent DVs for nutrients were truncated at 100, so that an excessively high intake of one nutrient could not compensate for the dietary inadequacy of another. In LIM, only the share in excess of the recommended amount was considered.

The development and validation of the NRF family of nutrient density indices have been reported in the literature (27). In the present adaptation, vitamin D, a nutrient of public health concern (28), replaced vitamin E. Fiber, vitamin D, calcium, magnesium, and potassium were all identified in the 2015 Dietary Guidelines for Americans as nutrients of concern (28). The NRF score was adjusted for energy intakes, analogous with the recent versions of the USDA Healthy Eating Index, a federal measure of diet quality (22). Both NRF9.3d and HEI 2015 were corrected for dietary energy (1,000 kcal for HEI and 2,000 kcal for NRF).

### Plan of Analysis

The sample was stratified by gender, age group, IPR (income-to-poverty ratio) and race/ ethnicity. Diet quality measures—Healthy Eating Index 2015 and the Nutrient Rich Food (NRF) index—were dichotomized to allow for comparisons between high quality diets and diets that needed work (22). Healthy diets were those in the top quartile of HEI 2015 and NRF scores. Weight status was measured using BMI z-scores that were split into 4 classes using clinically meaningful cut-points. We then conducted regression analyses between key indexes of consumption and BMI z-scores, adjusting for covariates.

Analyses of beverage consumption (g/day) were conducted for the entire population and by age group. All analyses accounted for the complex survey design of NHANES data and are representative of the US population. Data analyses were conducted using Stata 13.1 (College Station, TX) and SAS 9.4 (SAS institute, Cary, NC).

## Results

Table 1 shows participant characteristics. The sample of children and adolescents was evenly distributed by gender and income-to-poverty ratio (IPR). The sample was 52.1 % Non-Hispanic White; 14.6% Non-Hispanic Black, 15.7% Mexican American, and 8.0% other Hispanic.

**Table 1:** Sample characteristics by age group, demographics, diet quality and BMI z-scores.

	Total *N* = 9069		2–4y *N* = 1729	5–8y *N* = 2133	9–13y *N* = 2501	14–19y *N* = 2706	*p*-value
	*n*	%	%	%	%	%	
Gender							0.10
Male	4574	51.0	48.3	54.2	50.1	51.0	
Female	4495	49.0	51.7	45.8	49.9	49.0	
Family IPR							0.02
<1.3	2892	24.5	27.1	26.8	23.7	22.3	
1.3-1.849	2280	22.8	23.4	21.3	23.1	23.3	
1.85-2.99	1590	21.1	18.0	19.8	24.6	20.6	
≥3.0	1655	25.8	25.7	26.5	23.7	27.1	
Missing	652	5.7	5.8	5.5	4.8	6.6	
Race/ethnicity							0.58
Non-Hispanic White	2335	52.1	50.7	52.0	51.5	53.4	
Non-Hispanic Black	2345	14.6	15.2	14.5	13.6	15.1	
Mexican American	1925	15.7	15.2	15.9	15.9	15.7	
Other Hispanic	1032	8.0	8.7	8.5	8.6	6.9	
Non-Hispanic Asian	867	4.6	4.5	4.3	4.9	4.8	
Other	565	4.9	5.6	4.9	5.4	4.1	
BMI Z-score cutpoints							<0.001
Underweight	295	3.4	2.9	3.7	3.8	3.0	
Normal	5521	61.8	70.1	64.1	58.4	58.9	
Overweight	1443	15.7	13.6	14.4	17.4	16.1	
Obese	1645	17.6	9.5	17.5	19.9	19.8	
Missing	165	1.6	3.9	0.3	0.5	2.1	
HEI 2015							<0.001
Low (below Q3 = 56.58)	6760	75.0	60.6	74.0	78.7	79.7	
High (above Q3 = 56.58)	2309	25.0	39.4	26.0	21.3	20.3	
NRF9.3							<0.001
Low (below Q3 = 653.38)	6901	75.0	58.4	72.5	77.3	82.7	
High (above Q3 = 653.38)	2168	25.0	41.6	27.5	22.7	17.3	

*Data for children and adolescents, United States 2011-2016 NHANES*.

**Figure 1:**
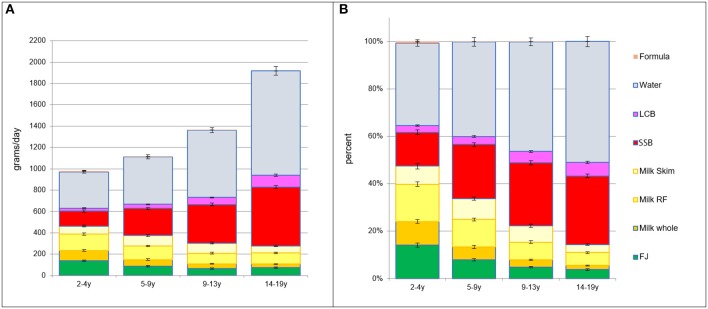
**(A)** Intakes in grams per day by beverage type and by age group. **(B)** Intakes in percent of daily intake by beverage type and by age group.

Beverages were separated into juice, milk (whole, reduced, skim), SSB, LCB, and water. Figure 1 shows beverage consumption (in g/d) by age group. Panel 1 (left) shows absolute intakes in g/d; panel 2 (right) shows proportions. The consumption of milk (all types) and juice declined sharply with age. Conversely, the consumption of water, SSB, and LCB increased with age. Absolute intake data presented in Figure 1 are also available in Supplemental Table 1.

**Figure 2:**
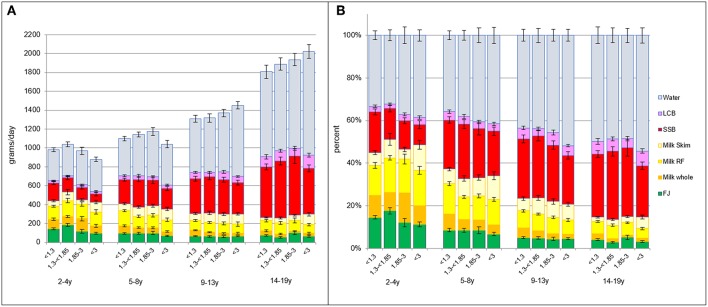
**(A)** Intakes in grams per day by beverage type and by age group and IPR strata. **(B)** Intakes in percent of daily intake by beverage type and by age group and IPR strata.

Beverage consumption patterns were linked to incomes, as expected. Figure 2 shows socioeconomic gradient in beverage consumption by 4 IPR strata, separately for each age group. There was a progressive drop in the consumption of whole milk at higher household incomes and a corresponding increase in consumption of reduced fat and skim milk. There was also an income associated drop in SSB consumption and an increase in LCB and water consumption. Consumption of juice was lower at higher IPR but only for young children (2–4y).

**Figure 3:**
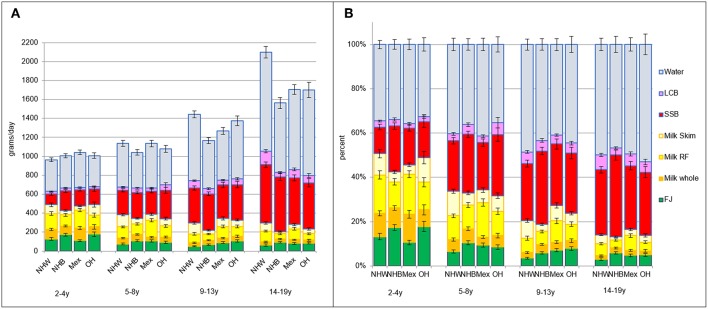
**(A)** Intakes in grams per day by beverage type and by age group and race/ethnicity. NHW, Non-Hispanic White; NHB, Non-Hispanic Black; Mex, Mexican American; OH, Other Hispanic. **(B)** Intakes in percent of daily intake by beverage type and by age group and race/ethnicity.

Figure 3 shows that beverage consumption patterns varied by race/ethnicity. Non-Hispanic White children drank more water, LCB, and skim milk than other racial/ethnic groups, especially after age 9 years. Non-Hispanic Black children drank more SSB, again especially later in childhood after 9 years.

### Diet Quality and Beverage Consumption

**Figure 4:**
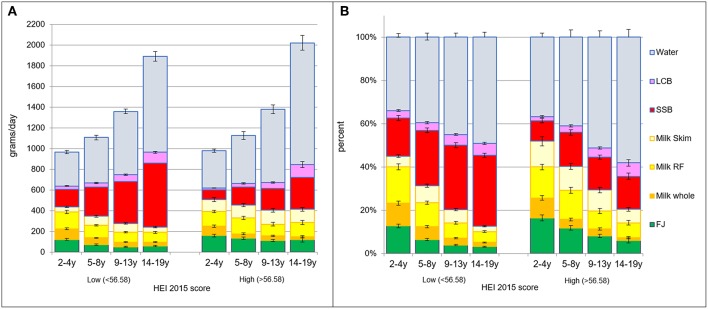
**(A)** Intakes in grams per day by beverage type and by age group and HEI quartile: lower (<56.58) vs. higher (>56.58). **(B)** Intakes in percent of daily intake by beverage type and by age group and HEI quartile: the lower (<56.58) vs. higher (>56.58).

Figure 4 shows beverage consumption patterns that were associated with the top quartile of HEI 2015 scores. Data are shown separately for each age group. The left panels show absolute intakes; right panels show proportions. Diets in the top quartile of HEI 2015 scores had more fruit juice, more reduced-fat and skim milk, and more drinking water. Lower quality diets were associated with higher SSB intakes.

**Figure 5:**
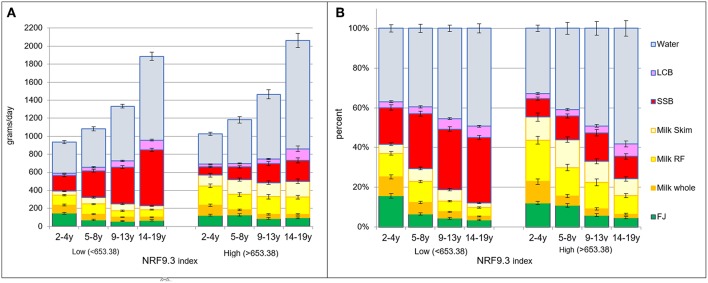
**(A)** Intakes in grams per day by beverage type and by age group and NRF quartiles: the lower (<653.38) vs. higher (>653.38). **(B)** Intakes in percent of daily intake by beverage type and by age group and NRF quartile: the lower (<653.38) vs. higher (>653.38).

The same pattern was observed using NRF9.3 as the measure of diet quality. Figure 5 shows beverage patterns associated with lower and higher NRF scores. Data are shown separately for each age group. The left panels show absolute intakes; right panels show proportions. Diets in the top quartile of NRF9.3 scores had more fruit juice, more skim and reduced-fat milk and more water. Lower quality diets were associated with higher SSB intakes.

### Beverage Consumption Patterns and Body Weight Status

Table 2 shows beverage consumption patterns as a function of BMI z-scores, separated into 4 classes: underweight, normal, overweight, and obese. There was no relation between fruit juice consumers and body weight status. There was no relation between milk consumption and body weight status. There was no significant relation between water consumption and body weight status.

**Table 2:** Average amount (g/d) of fruit juices, milk, and water by BMI class, in the total NHANES sample.

		**Fruit juices**				**Milk**				**Water**			
**BMI classes**	***N***	**Mean**	**SE**	**Lower limit**	**Upper limit**	**Mean**	**SE**	**Lower limit**	**Upper limit**	**Mean**	**SE**	**Lower limit**	**Upper limit**
Underweight	295	70.30	15.57	38.98	101.62	265.65	36.11	193.01	338.29	536.36	125.47	283.94	788.78
Normal	5521	88.01	4.27	79.42	96.60	259.38	8.66	241.97	276.80	636.51	24.04	588.16	684.87
Overweight	1443	85.70	5.53	74.58	96.81	237.47	8.44	220.49	254.44	667.70	33.92	599.46	735.95
Obese	1645	72.10	6.80	58.41	85.78	235.57	9.08	217.30	253.83	758.78	36.83	684.69	832.87
Missing	165	122.34	32.61	56.72	187.95	212.21	31.68	148.48	275.94	673.41	86.55	499.30	847.52

To test for interactions, additional analyses were conducted with BMI classes as independent variables and fruit juice, milk, and water as dependent variables, adjusting for gender and age group, poverty level, and ethnicity. The age^*^BMI interaction was also included in the general linear model to test whether any interaction between beverage consumption and BMI classes was age dependent. No significant interactions were observed.

Table 3 shows percent compliance with the AAP recommendations for juice consumption, separately for each age group. Dietary compliance was calculated separately for each age group according to AAP standards. For 1–3 year-olds, the standard was <=2 oz juice per day; for 4–6 year-olds, it was between 4 and 6 ounces per day, and for 7–18 year-olds it was <8 ounces per day.

**Table 3:** Compliance with American Academy of Pediatrics (AAP) recommendations for fruit juice by gender, diet quality and body weight status.

	Total *N* = 9069		2–4y *N* = 1729	5–8y *N* = 2133	9–13y *N* = 2501	14–19y *N* = 2706
	*n*	%	%	%	%	%
**GENDER**						
Male	4574	68.0	38.1	48.7	83.3	82.8
Female	4495	71.4	44.9	50.0	86.0	85.8
*p*-value		0.03	0.05	0.66	0.12	0.15
**HEI 2015**						
Low (below Q3 = 56.58)	6760	73.4	44.0	49.8	88.0	86.7
High (above Q3 = 56.58)	2309	58.4	38.0	47.8	72.1	74.8
*p*-value		<0.001	0.06	0.55	<0.001	<0.001
**NRF9.3**						
Low (below Q3 = 653.38)	6901	72.4	39.8	50.4	86.4	85.6
High (above Q3 = 653.38)	2168	61.2	44.2	46.5	78.6	77.9
*p*-value		<0.001	0.14	0.35	0.008	0.0002
**BMI Z-SCORE CUTPOINTS**						
Underweight	295	77.5	52.6	70.4	82.7	89.0
Normal	5521	68.1	42.8	47.4	85.7	83.4
Over weight	1443	69.6	36.0	48.0	84.1	83.2
Obese	1645	73.3	35.0	52.9	82.2	86.7
Missing	165	70.2	47.7	51.2	88.9	88.8
*p*-value		0.02	0.18	0.11	0.64	0.45

*Data are shown separately for each age group*.

The data are split by gender, diet quality and BMI z-scores. First, diets of about 70% of the children were consistent with the AAP recommendations. Compliance rates ranged for 38% to 85% depending on age-specific consumption limits set by AAP. Interestingly, higher quality diets, assessed using two separate diet quality scores, were associated with lower compliance with AAP guidelines for juice. There was no relation between diets consistent with AAP recommendations and the children's body weight status.

## Discussion

Based on a representative sample of US toddlers, children, and adolescents aged 2–19 years, the present analyses include the most recent NHANES 2015-16 data. The comparisons made were between milk, juice and drinking water. We have previously identified beverage patterns built around milk and juice as providing an optimum selection of nutrients—from vitamin C to calcium and protein (4). However, few children in the NHANES 2011-14 dataset drank mostly milk and juice. The vast majority had beverage drinking patterns built around SSB.

In the present study, diets in the top quartile of HEI 2015 and NRF9.3 scores had more milk, more juice, and more water. By contrast, lower quality diets had more SSB.

We also confirm the previously observed age effect—whereas milk and juices sharply decline with age, SSB and water increase. The age-related shifts in beverage choice can have consequences for nutrient density of the diet. Preventing the shift to SSB by continuing to drink milk and juice can confer some nutritional advantage.

Previously observed social gradients in beverage consumption were also confirmed in the present analyses. As expected, children from households with higher SES groups drank more skim and reduced fat milk and more water. By contrast, children from households with lower SES drank more SSB and more whole milk. A similar relationship for race/ethnicity was observed where Non-Hispanic White children drank more water, LCB, and skim milk and NHB children drank more SSB.

Previous studies showed that the consumption of whole fruit vs. fruit juice was income driven. Whereas, higher SES groups consumed more whole fruit, lower SES groups consumed more juice. Those studies also indicated that whole fruit accounted for 66% of total fruit consumption; there was no indication that juice displaced whole fruit in any way (29).

The present analyses showed no association between juice consumption and BMI z-scores, in line with a recent review concluded that there is not enough evidence to support the association of fruit juice intake and weight status or adiposity (2). A recent meta-analysis found that consumption of fruit juice lead to small, not clinically significant weight gain in children aged 1–6 years and no weight gain in older children (7–18 years). The researchers suggest that the type of fruit juice consumed may account for this age difference; where younger children tend to drink apple juice and older children are more likely to consume orange juice, which may have different effects on cardiometabolic health due to differences in glycemic load (16). Similar analyses were conducted for whole, reduced fat, and skim milks.

Only about 30% of children drank juice on the first day of NHANES data collection. Although beverage drinking patterns built around milk and juice are most nutrient rich, most children drink SSB. Furthermore, both milk and juice consumption drop with age, whereas the consumption of SSB and water increases. Replacing milk and juice with water is one public health goal—however, milk and juice are also being replaced with SSB. Promoting milk and juice consumption into early adolescence may have some nutritional advantages.

The present analyses incorporate one of the few analyses of how compliance with the AAP juice guidelines related to diet quality overall. First, compliance with the AAP guidelines was lower for younger children who drank for juice than older children. Older children drank less juice and were therefore more compliant with the AAP guidelines; however, those children also drank more SSB. Paradoxically, better compliance with AAP guidelines (i.e., less juice) was associated with lower dietary nutrient density scores (i.e., more SSB). Whereas, replacing juice with plain water may be the desirable public health goal, the fact is that both water and SSB consumption increase with age.

Second, there was no association between compliance with AAP recommendations and body weight status. Together these novel results, albeit based on cross-sectional data, suggest that limiting juice intake may not have the intended effect of reducing obesity risk and may be at odds with other measures of diet quality.

The present study had limitations. Most important, the NHANES data were cross sectional, meaning that no causal inferences can be drawn. The present discussion is therefore limited to associations or lack thereof. Dietary intakes data were self-reported, and included proxy report for children 5 years or younger. There are limits to the accuracy of the self-reported data, such that participants are likely not able to report specific details of their intake; i.e., whether a juice was pasteurized or sterilized. Thus, the USDA nutrient composition database only classifies juice into the following categories; freshly squeezed, canned, bottled, frozen reconstituted from concentrate. In addition, self-reported dietary data are always subject to under- and over-reporting. However, the NHANES data are thoroughly examined in several rounds by trained reviewers before being approved for usage, ensuring high quality data (30). Finally, due to the cross-sectional design of the NHANES study, causality cannot be inferred from the data. Despite these limitations, NHANES data are still used as the basis for dietary policies in the US. Nutrient profiling models of nutrient density are nutrient based and may not adequately capture multiple aspects of healthy food patterns. The same limitation applies to the food and nutrient based Healthy Eating Index 2015. Though it is the preferred USDA measure of compliance with the 2015 USDA dietary guidelines, it may not adequately capture food patterns across the social strata.

## Conclusions

Beverage consumption patterns varied with age; older children replaced milk and juice with SSB and to some extent with water. Higher SSB consumption was associated with lower quality diets. Lower compliance with AAP recommendations was associated with higher quality diets. While the current dietary guidance is for children to replace milk and juice with plain water, there is a high likelihood that children will drink palatable SSB instead. Finally, the AAP recommendation to limit juice intake were not associated with weight status, suggesting that compliance with the recommendations may have limited value for obesity prevention.

## Data Availability

The datasets for this study will not be made publicly available because Federal data from the NHANES study are already publicly available can be accessed here: https://wwwn.cdc.gov/nchs/nhanes/default.aspx.

## Ethics Statement

The ethics board review for the NHANES data collection is documented by the National Center for Health Statistics online (20). Analyses of publicly available federal NHANES data are exempt from approvals by Institutional Review Boards.

## Author Contributions

MM, FV, CR, and AD designed the study. CR developed the databases. MM and FV conducted the analyses. AD took the lead on writing the paper, along with CMR. All authors read and approved the final manuscript.

### Conflict of Interest Statement

AD has received grants, contracts, and honoraria from numerous entities, both public and private, for studies on dietary nutrient density and nutrient profiling of individual foods and food patterns. FV and MM are employed by MS-Nutrition, a consulting firm. The remaining authors declare that the research was conducted in the absence of any commercial or financial relationships that could be construed as a potential conflict of interest.

## References

[1] Clemens R Drewnowski A Ferruzzi MG Toner CD Welland D. Squeezing fact from fiction about 100% fruit juice. Adv Nutr. (2015) 6:236S−243S. 10.3945/an.114.007328 25770266 PMC4352186

[2] Crowe-White K O'Neil CE Parrott JS Benson-Davies S Droke E Gutschall M. Impact of 100% fruit juice consumption on diet and weight status of children: an evidence-based review. Crit Rev Food Sci Nutr. (2016) 56:871–84. 10.1080/10408398.2015.1061475 26091353

[3] Heyman MB Abrams SA. Fruit juice in infants, children, and adolescents: current recommendations. Pediatrics. (2017) 139:e20170967. 10.1542/peds.2017-0967 28562300

[4] Maillot M Rehm CD Vieux F Rose CM Drewnowski A. Beverage consumption patterns among 4–19 y old children in 2009–14 NHANES show that the milk and 100% juice pattern is associated with better diets. Nutr J. (2018) 17:54. 10.1186/s12937-018-0363-9 29793492 PMC5968613

[5] Murphy MM Douglass JS Johnson RK Spence LA Drinking flavored or plain milk is positively associated with nutrient intake and is not associated with adverse effects on weight status in US children and adolescents. J Am Diet Assoc. (2008) 108:631–9. 10.1016/j.jada.2008.01.004 18375219

[6] Byrd-Bredbenner C Ferruzzi MG Fulgoni VL III Murray R Pivonka E Wallace TC. Satisfying America's fruit gap: summary of an expert roundtable on the role of 100% fruit juice. J. Food Sci. (2017) 82:1523–34. 10.1111/1750-3841.13754 28585690

[7] Gidding SS Dennison BA Birch LL Daniels SR Gilman MW Lichtenstein AH. Dietary recommendations for children and adolescents: a guide for practitioners. Pediatrics. (2006) 117:544–59. 10.1542/peds.2005-2374 16452380

[8] Baker SS Cochran WJ Greer FR Heyman MB Jacobson MS Jaksic T The use and misuse of fruit juice in pediatrics. Pediatrics. (2001) 107:1210–3. 10.1542/peds.107.5.1210 11331711

[9] Drewnowski A Buszkiewicz J Aggarwal A. Soda, salad, and socioeconomic status: Findings from the Seattle Obesity Study (SOS). SSM-Popul Health. (2019) 7:100339. 10.1016/j.ssmph.2018.100339 30623013 PMC6317301

[10] Kit BK Carroll MD Ogden CL. Low-Fat Milk Consumption Among Children and Adolescents in the United States, 2007–2008. Hyattsville, MD: National Center for Health Statistics (2011). 22617139

[11] Rehm CD Drewnowski A. Dietary and economic effects of eliminating shortfall in fruit intake on nutrient intakes and diet cost. BMC Pediatr. (2016) 16:83. 10.1186/s12887-016-0620-z 27387744 PMC4937591

[12] Dubowitz T Heron M Bird CE Lurie N Finch BK Basurto-Dávila R. Neighborhood socioeconomic status and fruit and vegetable intake among whites, blacks, and Mexican Americans in the United States. Am J Clin Nutr. (2008) 87:1883–91. 10.1093/ajcn/87.6.1883 18541581 PMC3829689

[13] Drewnowski A Rehm CD Constant F. Water and beverage consumption among children age 4-13y in the United States: analyses of 2005–2010 NHANES data. Nutr J. (2013) 12:85. 10.1186/1475-2891-12-85 23782914 PMC3698018

[14] Drewnowski A Rehm CD Constant F. Water and beverage consumption among adults in the United States: Cross-sectional study using data from NHANES 2005–2010. BMC Publ Health. (2013) 13:1068. 10.1186/1471-2458-13-1068 24219567 PMC3840570

[15] Ogden CL Lamb MM Carroll MD Flegal KM Obesity and Socioeconomic Status in Children and Adolescents: United States, 1988–1994 and 2005–2008. NCHS Data Brief. Number 51. Hyattsville, MD: National Center for Health Statistics (2010).

[16] Auerbach BJ Wolf FM Hikida A Vallila-Buchman P Littman A Thompson D. Fruit juice and change in BMI: a meta-analysis. Pediatrics. (2017) 139:e20162454. 10.1542/peds.2016-2454 28336576 PMC5369671

[17] Darmon N Drewnowski A. Contribution of food prices and diet cost to socioeconomic disparities in diet quality and health: a systematic review and analysis. Nutr Rev. (2015) 73:643–60. 10.1093/nutrit/nuv027 26307238 PMC4586446

[18] Del Valle HB Yaktine AL Taylor CL Ross AC Dietary Reference Intakes for Calcium and Vitamin D. National Academies Press (2011). 21796828

[19] Hamner HC Perrine CG Gupta PM Herrick KA Cogswell ME Dietary patterns among children birth to 23 months of age, NHANES 2009-2014. J Nutr Edu Behav. (2017) 49:S1 10.1016/j.jneb.2017.05.010 PMC5622702 28846605

[20] Centers for Disease Control and Prevention NCHS Research Ethics Review Board (ERB) Approval. (2017). Available online at: https://www.cdc.gov/nchs/nhanes/irba98.htm (accessed on April 17, 2019).

[21] Bowman SA Clemens JC Shimizu M Friday JE Moshfegh AJ Food Patterns Equivalents Database 2015–2016: Methodology and User Guide [Online]. Beltsville, MD: Food Surveys Research Group, Beltsville Human Nutrition Research Center, Agricultural Research Service, U.S. Department of Agriculture (2018). Available online at: http://www.ars.usda.gov/nea/bhnrc/fsrg

[22] Krebs-Smith S Pannucci T Subar A Kirkpatrick S Lerman J Tooze J. (2018). Update of the healthy eating index: HEI-2015. J Acad Nutr Diet. 118:1591–602. 10.1016/j.jand.2018.05.021 30146071 PMC6719291

[23] Drewnowski A Rehm C Vieux F Breakfast in the United States: food and nutrient intakes in relation to diet quality in NHANES 2011-2014. A study from the international breakfast research initiative (IBRI). Nutrients. (2018) 10:E120 10.20944/preprints201808.0109.v1 30200424 PMC6163505

[24] Drewnowski A. The Nutrient rich foods index helps to identify healthy, affordable foods–. Am J Clin Nutr. (2010) 91:1095S–1101S. 10.3945/ajcn.2010.28450D 20181811

[25] Francou A Hebel P Braesco V Drewnowski A. Consumption patterns of fruit and vegetable juices and dietary nutrient density among french children and adults. Nutrients. (2015) 7:6073–87. 10.3390/nu7085268 26213964 PMC4555108

[26] Rehm CD Drewnowski A. Replacing American breakfast foods with ready-to-Eat (RTE) cereals increases consumption of key food groups and nutrients among US children and adults: results of an NHANES modeling study. Nutrients. (2017) 9:1010. 10.3390/nu9091010 28902145 PMC5622770

[27] Drewnowski A Fulgoni VL III. Nutrient density: principles and evaluation tools–. Am J Clin Nutr. (2014) 99:1223S–8S. 10.3945/ajcn.113.073395 24646818

[28] U.S. Department of Health and Human Services and U.S. Department of Agriculture. 2020 Dietary Guidelines for Americans, 8th ed (2015). Available online at: https://health.gov/dietaryguidelines/2015/guidelines/ (accessed on April 17, 2019)

[29] Drewnowski A Rehm CD. Socioeconomic gradient in consumption of whole fruit and 100% fruit juice among US children and adults. Nutr J. (2015) 14:3. 10.1186/1475-2891-14-3 25557850 PMC4326504

[30] Ahluwalia N Dwyer J Terry A Moshfegh A Johnson C. Update on NHANES dietary data: focus on collection, release, analytical considerations, and uses to inform public policy. Adv Nutr. (2016) 7:121–34. 10.3945/an.115.009258 26773020 PMC4717880

