# Supplementary material for: Consumption Patterns of Milk and 100% Juice in Relation to Diet Quality and Body Weight Among United States Children: Analyses of NHANES 2011-16 Data
Source: Front Nutr. 2019 Aug 8;6:117. doi: 10.3389/fnut.2019.00117 (PMC6694734; doi:10.3389/fnut.2019.00117)
Supplement: Supplementary file 1 [file Table_1.docx]

**Supplemental Table 1:**

**Beverage consumptions (in g/d) by age group**

|  | **2-4y** | | **5-8y** | | **9-13y** | | **14-19y** | | **pvalue** |
| --- | --- | --- | --- | --- | --- | --- | --- | --- | --- |
| N | 1729 | | 2133 | | 2501 | | 2706 | |  |
| **Food groups, g/d** | **Mean** | **SE** | **Mean** | **SE** | **Mean** | **SE** | **Mean** | **SE** |  |
| 100% Fruit juices | 138.1 | 8.1 | 87.7 | 5.6 | 65.4 | 4.5 | 72.6 | 7.2 | <.0001 |
| Milks (all) | 325.8 | 11.9 | 286.8 | 9.9 | 238.4 | 9.3 | 202.6 | 9.3 | <.0001 |
| Whole milk | 97.3 | 7.7 | 60.4 | 4.3 | 43.7 | 3.6 | 34.5 | 3.8 | <.0001 |
| Reduced fat milk | 153.9 | 9.7 | 129.2 | 6.6 | 100.0 | 8.2 | 103.3 | 7.0 | 0.0002 |
| Low fat/skim milk | 74.6 | 10.7 | 97.2 | 7.8 | 94.8 | 8.4 | 64.8 | 6.2 | 0.002 |
| Infant formula | 5.8 | 1.5 | 1.3 | 0.5 | 0.3 | 0.1 | 0.0 | 0.0 | <.0001 |
| Other LC beverages | 27.6 | 3.2 | 38.0 | 4.8 | 66.0 | 5.4 | 110.2 | 9.9 | <.0001 |
| Other HC beverages | 139.9 | 9.0 | 255.0 | 8.7 | 362.0 | 12.5 | 554.5 | 14.6 | <.0001 |
| Water | 340.5 | 12.5 | 443.3 | 19.8 | 630.9 | 22.7 | 976.4 | 41.0 | <.0001 |
